# Effect of Regulator of G Protein Signaling Proteins on Bone

**DOI:** 10.3389/fendo.2022.842421

**Published:** 2022-04-29

**Authors:** Gongsheng Yuan, Shuying Yang

**Affiliations:** ^1^ Department of Basic and Translational Sciences, Penn Dental Medicine, University of Pennsylvania, Philadelphia, PA, United States; ^2^ The Penn Center for Musculoskeletal Disorders, Penn Medicine, University of Pennsylvania, Philadelphia, PA, United States; ^3^ Center for Innovation and Precision Dentistry, Penn Dental Medicine, School of Engineering and Applied Sciences, University of Pennsylvania, Philadelphia, PA, United States

**Keywords:** bone homeostasis, osteoclast (OC), osteoblast (OB), chondrocyte, GPCR (G protein coupled receptor), bone development, regulator of G protein signaling (RGS)

## Abstract

Regulator of G protein signaling (RGS) proteins are critical negative molecules of G protein-coupled receptor (GPCR) signaling, which mediates a variety of biological processes in bone homeostasis and diseases. The RGS proteins are divided into nine subfamilies with a conserved RGS domain which plays an important role in regulating the GTPase activity. Mutations of some RGS proteins change bone development and/or metabolism, causing osteopathy. In this review, we summarize the recent findings of RGS proteins in regulating osteoblasts, chondrocytes, and osteoclasts. We also highlight the impacts of RGS on bone development, bone remodeling, and bone-related diseases. Those studies demonstrate that RGS proteins might be potential drug targets for bone diseases.

## Introduction

Bone homeostasis is dynamically maintained by the processes of bone formation and resorption, which initiates with the osteoclasts-derived resorption of the calcified bone matrix and is followed by the osteoblast-regulated bone formation (1, 2). Any malfunctions in osteoclast and/or osteoblast formation will cause changes in the bone mass and defective skeletal integrity (3). In addition, chondrocytes contribute to skeletal development through endochondral ossification, abnormality of chondrocyte formation and function can also cause bone-related diseases such as bone dysplasia and osteoarthritis (4).

G protein-coupled receptors (GPCRs) are located in the cell membrane that transmit signals into the intracellular environment (5, 6). The GPCRs are activated by extracellular substances and further promote the exchange of Gα (6). Gα dissociates from the Gβγ dimer by transiting to the active GTP form from its inactive GDP form (7). The active GTP-bound Gα and Gβγ further trigger the downstream signaling pathways (7). RGS proteins are the main negative regulators in GPCR regulatory pathways (8). Those proteins negatively modulate the G protein signaling through the stimulation of Gα-mediated GTP hydrolysis (9). Studies in human patients have shown that changes in some RGS proteins are associated with numerous complex polygenic pathologies including hypertension, atherosclerosis, cancers, immune disorders, heart and brain diseases (10–17). Those findings are further validated in gene conventional and conditional knockout animal models. Moreover, the impacts of RGS proteins on a variety of GPCR signaling pathways have been determined in those animal models (9, 18). Up to now, the RGS family members have been divided into nine subfamilies including RZ (RGS17, 19, 20), R4 (RGS1-5, 8, 13, 16, 18, 21), R7 (RGS6, 7, 9, 11), R12 (RGS10, 12,14), RA (AXIN, AXIN2), GEF (p115-RhoGEF, PDZ-RhoGEF, LARG), GRK (GRK1-7), SNX (SNX13, 14, 25), and others (RGS22, D-AKAP2) (19). Some RGS proteins and their signaling pathways have been discovered to regulate skeletal formation/remodeling, and the changes in these RGS proteins leads to various bone diseases in human. In this review, we summarized the recent research advances regarding RGS proteins in the regulation of bone.

## Role of RGS Proteins in Bone

**Figure 1:**
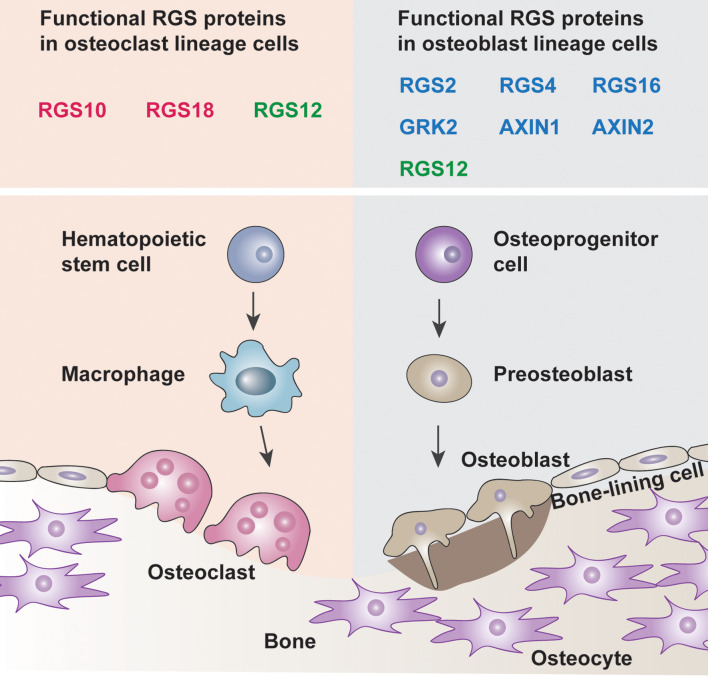
Roles of RGS proteins in bone cells. Osteoblast lineage cells drive bone development whereas the osteoclast lineage cells promote bone remodeling. The balance between the osteoblasts and osteoclasts leads to bone homeostasis. The box indicated the known functional RGS proteins are expressed in the osteoclast lineage cells and osteoblast lineage cells (Red, osteoclast solely, Blue, osteoblast solely, Green, both osteoclast and osteoblast).

RGS proteins are widely associated with bone development and remodeling under physiological and pathological conditions (10, 20, 21). Osteoblasts and osteoclasts are the two main constituents that can be affected by RGS proteins (**Figure 1**). Studies have shown that a group of RGS proteins such as RGS2, RGS4, RGS16, AXIN1, AXIN2, and G protein-coupled receptor kinase 2 (GRK2) are mainly expressed in osteoblasts or osteoprogenitor cells (22–27), while RGS10 and RGS18 are mainly expressed in osteoclasts and macrophages (28, 29). Interestingly, RGS12 is expressed in both osteoblasts and osteoclasts according to our findings (**Table 1**) (35, 37). Since several RGS protein functions have been summarized in detail in previous publications, in this review, we mainly focused on summarizing the latest publications on RGS proteins in bone cells including osteoblasts, osteoclasts, chondrocytes as well as animal bone phenotypes, and diseases.

**Table 1:** The impact of RGS proteins on bone homeostasis.

Member	Family	Gα GAP activity	Additional domain(s)	Bone cell type and function	Potential association with GPCRs in bone	REF
RGS2	R4	Gαq	AH	Osteoblast differentiation	PTH1R	(10, 23, 30)
RGS4	R4	Gαi/o and Gαq/11	AH	Osteoblast differentiation	GPRC6A	(10, 23, 31)
RGS5	R4	Gαi/o and Gαq/11	AH	Chondrocyte differentiation	PTH1R	(10, 32, 33)
RGS10	R12	Gαi/o and Gαq/11		Osteoclast differentiation	CasR, OGR1	(10, 28, 30, 34)
				Chondrocyte differentiation	N/A	(32)
RGS12	R12	Gαi/o	PDZ, PTB, RBD, GoLoco	Osteoclast differentiation	CasR, OGR1	(10, 21, 30, 35, 36)
				Osteoblast differentiation, maturation	N/A	(37)
				Chondrocyte maturation	N/A	(38)
RGS16	R4	Gαi/o and Gαq/11	AH	Osteoblast differentiation	OGR1	(24)
RGS18	R4	Gαi/o and Gαq/11	AH	Osteoclast differentiation	CasR, OGR1	(10, 29, 30)
Axin1	RA	N/A	CC, DAX, GSK3β BD. β-catenin BD	Osteoblast differentiation	N/A	(25, 30)
Axin2	RA	N/A	CC, DAX	Osteoblast differentiation	N/A	(26, 39–41)
GRK2	GRK	N/A	S/T kinase, PH, CC	Osteoblast differentiation	PTHrP	(27, 42)

AH, amphiphatic helix; S/T kinase, serine/threonine kinase domain; PH, pleckstrin homology domain; PDZ, domain present in PSD-95; PTB, phosphotyrosinebinding domain; β-catenin BD, β-catenin binding domain; RBD, Raf-like Ras; GoLoco, G protein regulatory motif; DAX, domain present in disheveled and axin; GSK3β BD, GSK3β-binding domain; CC, coiled coil motif; PTH1R, parathyroid hormone 1 receptor; CasR, calcium-sensing receptor; OGR1, ovarian cancer G protein-coupled receptor 1; PTHrP, Parathyroid hormone-related peptide; REF, reference; N/A, not applicable.

## RGS Proteins in Osteoblasts

A few RGS proteins have been recently reported to modulate the functions of osteoblasts and bone formation (30). RGS12 is the largest protein in RGS protein family, which is highly expressed in osteoblasts and gradually increased during osteogenesis (37). Our laboratory found that the loss of RGS12 in osteoblasts precursor cells leads to decreased osteoblast differentiation, maturation, and mineralization. Mechanistically, the loss of RGS12 in osteoblast precursors represses the activation of GTPase and impairs the main sources of calcium entry through the blockage of L-type calcium channel (37). RGS16 is a conserved protein in R4 RGS subfamily. RGS16 is also a peripheral membrane protein, which showed high transcript expression in osteoblasts from calvaria bone (24). Interestingly, the RGS16 mRNA expression of calvarial osteoblasts is decreased in the acid medium, which prolongs the response of ovarian cancer G protein coupled receptor 1 (OGR1) to stimulate bone erosion (24). This study suggested that osteoblastic RGS16 plays a critical role in regulating the response of OGR1 to metabolic acidosis and subsequent bone resorption (24). AXIN2 has been identified to dominantly express in the suture stem cells from calvarial bone and act as a key player in skeletal formation (39). The knockout of AXIN2 promotes the downstream Rap1b expression through activation of canonical bone morphogenetic protein (BMP) signaling, which is critical for craniofacial bone development (39). Consistently, the global AXIN2 KO mice increased osteoblast functions by indicating enhanced mineral appositional rates (MAR) and bone formation rates (BFR) in 6- and 12-month-old mice (26). Similarly, the loss of AXIN2 in periodontal ligament progenitor cells results in a significant decline in osteogenic activity (as reflected by ALP) in alveolar bone (40). Thus, recent findings demonstrate that RGS12, RGS16, and AXIN2 are major players in osteoblasts, which enriches the theory of the regulation of osteogenesis by RGS proteins.

## RGS Proteins in Chondrocytes

Chondrogenesis mainly contributes to the production of hypertrophic chondrocytes and initiation of subchondral bone formation (43). RGS proteins have been found to play an important role during chondrogenesis (32). RGS10 in chondrocytes can promote the expression of earlier markers such as Col2a1 and Sox9 (32, 44). Moreover, RGS10 induces chondrogenic differentiation through increasing glycosaminoglycans (GAG) synthesis, alkaline phosphatase (ALP) activity, and Col10 expression (32). Similarly, the overexpression of RGS5 in chondrocytes can also enhance the GAG synthesis, cell proliferation, and PTHrP-induced cAMP levels (32, 33). Moreover, RGS5 induces the expression of Indian hedgehog (Ihh, an early marker of post-mitotic chondrocytes) to stimulate chondrogenesis (32). Besides these RGS proteins, we recently found that RGS12 is located in the mitochondria of primary chondrocytes and controls chondrocyte homeostasis. The knockout of RGS12 leads to decreased mitochondrial functions as reflected by the decreased number of mitochondria, mitochondrial membrane potential, and increased apoptosis and cell death. We also found that RGS12 enhances the phosphorylation of ATP5 to further affect the mitochondrial functions and maturation of chondrocytes. Due to the mitochondrial dysfunction, the RGS12 conditional knockout mice in chondrocytes resulted in abnormal endochondral ossification and bone defects (38). Thus, RGS10, RGS5, and RGS12 have been demonstrated to mainly regulate the function of chondrocytes.

## RGS Proteins in Osteoclasts

Osteoclasts are regulated by numerous GPCRs such as optomotor-blind-related gene-1 protein (ORG1) and calcitonin receptors, which play critical roles in skeletal development (45–47). During the last ten years, RGS10, RGS12, and RGS18 have been reported to be involved in osteoclast differentiation and/or function as reviewed in reference (30). Our recent studies explored the function of RGS12 in regulating inflammation mediated osteolysis. We found that RGS12 was expressed at the highest level in monocytes cells compared with other immune cells in the blood, suggesting that it may have an important regulatory effect on the function of monocytes (48). The conditional knockout of RGS12 in monocytes/macrophages led to increased bone mass accompanied by decreased osteoclast number and activity but no alteration in osteoblast number or bone formation activity. Conversely, the forced overexpression of RGS12 resulted in the upregulation of osteoclast numbers and bone resorption activity. By analyzing the LC/MS data in RGS12 deficient osteoclasts, we identified the Nrf2, a major regulator of oxidative stress, was controlled by RGS12. Moreover, we found the RGS12 promoted the degradation of Nrf2 by activating 26S proteasome and further activated the RANKL-induced phosphorylation of ERK1/2 and NF-κB (36). Thus, our study discovered a novel signaling pathway of RGS12 in controlling the cellular redox and osteoclast functions.

## Effects of RGS Proteins in Bone Homeostasis

Previous studies regarding the regulation of RGS proteins in bone have been reviewed by Keinan et al. (30). RGS proteins were selected expressed in different tissues, RGS4, 7, 8, 11, and 17 were expressed in brain, RGS5 was mainly expressed in heart, and RGS1 was highly enriched in lung (49). According to previous studies, only few of RGS subfamilies including R4 (RGS2,4,5,16,18), R12 (RGS10,12), RA (AXIN1,2), and GRK (GRK2) were reported to express in bone (**Table 1**) (10, 30, 42). Here, we summarize the latest findings of RGS protein functions and regulatory mechanisms in bone homeostasis.

RGS2 and RGS5 proteins belong to RGS R4 subfamily. A study recently reported that RGS2 is expressed in rat cortical bone and mouse calvarial bone (30). Additionally, Koh et al. found that PTH also can induce RGS5 expression and activate the calcium-sensing receptor (CASR) in responses to extracellular calcium, and ablation of RGS5 in mice down-regulates PTH plasma levels (50). By creating the transgenic mouse with overexpression of RGS5 in the parathyroid gland, the authors found that these mice showed hyperparathyroidism and increased bone mass. Further, the forced overexpression of RGS5 in parathyroid cells impaired CASR signaling and negatively feedback on PTH secretion, demonstrating that RGS5 plays a critical role in bone formation through regulating PTH and CASR signaling (51).

AXIN1 and AXIN2 belong RA subfamily of RGS, which regulate bone development through activating beta-catenin signaling (26, 52). In Osx-Cre;AXIN1^fl/fl^ cKO mice, osteoclasts in the bone marrow cavity, ossification front, and subchondral bone are significantly reduced due to the increased osteoprotegerin (OPG) expression. AXIN2 global knockout mice display increased bone mass and mechanical strength due to the increased osteoblast differentiation and decreased osteoclast differentiation (26, 41). Further, AXIN2 expressing cells from tibia of AXIN2CreER;R26mTmG transgenic mice can promote bone regeneration after skeletal injury (53).

RGS10 and RGS12 belong to R12 subfamily of the RGS proteins. RGS10 knockout mice exhibit a serious osteopetrotic phenotype as a consequence of dysfunctional osteoclasts through impaired calcium oscillations/NFATc1 signaling pathway (28). Compared to RGS10, conditional knockout of RGS12 in osteoclast lineage (CD11b-Cre and Mx1-Cre) also caused osteopetrosis phenotype. However, the mechanisms regulated by RGS10 and RGS12 are different. The former associates with calcium/calmodulin and PIP3 in an intracellular calcium-dependent manner in osteoclasts and the latter controls calcium oscillations by facilitating calcium influx and elevating Nrf2/Keap1 expression to enhance osteoclast differentiation and activity (28, 36, 54, 55). These studies demonstrate that different RGS proteins play unique roles in bone homeostasis through regulating different signaling pathways.

## RGS Proteins in Bone-Related Diseases

Several studies have demonstrated that mutation or malfunction of RGS proteins can cause or contribute to bone-related diseases (30). The study by Li et al. found that LINC00370 (Long Intergenic Non-Protein Coding RNA 370) and RGS4 are both upregulated in osteogenic induction adipose-derived stem cells. LINC00370 acts as a sponge that can inhibit the expression of miR-222-3p, which further upregulates RGS4 expression, osteoblast differentiation, and prevents ovariectomized (OVX)-induced osteoporosis (31). Different from RGS4, the deletion of RGS10 or RGS12 in osteoclasts causes osteopetrosis phenotype through controlling the calcium oscillations and oxidative stress mediated Nrf2/Keap1 signaling pathways in mice, indicating their positive regulation in aging mediated osteoporosis (28, 55). These findings provide new strategies that targeting RGS proteins may be promising to improve bone mass and strength in osteoporosis patients.

Moreover, RGS proteins are also involved in inflammation mediated bone diseases. Periodontitis and arthritis are inflammatory diseases that mostly affect both immunity and bone homeostasis (56, 57). The study by Zhang et al. demonstrated that the inhibition of AXIN1 increases bone formation and reduces the inflammatory cytokine and the osteoblastic apoptosis triggered by porphyromonas gingivalis lipopolysaccharide (58). Yang et al. report that the decreased RGS10 can prevent inflammation and osteoclasts mediated bone erosion in bacteria-induced inflammatory lesions (34). Similarly, the conditional knockout of RGS12 in macrophages can prevent osteoclast differentiation and M1 macrophage polarization and activation in ligature-induced periodontitis mouse models (48). As for arthritis studies, RGS proteins regulate immune responses through several signaling pathways. For example, Hu et al. demonstrated that the inhibition of RGS1 can prevent inflammation and angiogenesis in rheumatoid arthritis through suppressing Toll-like receptor signaling (59). Interestingly, we have demonstrated that RGS12 can directly interact with NF-κB through its PTB domain to activate inflammatory responses in rheumatoid arthritis (21, 60). In addition, the loss of RGS12 in macrophages can inhibit osteoarthritis progression by decreasing the ubiquitination levels, which further inhibit the degradation of IκB (16). These studies suggest that the RGS proteins may have multiple functions, which are involved in the regulation of not only bone cells, but also immune cells mediated osteolysis.

## Conclusion and Perspectives

RGS proteins are critical for bone homeostasis by regulating not only the GPCR signaling pathways but also other important pathways including calcium signaling, BMP signaling, Indian hedgehog signaling, NF-κB signaling, Keap1-Nrf2 signaling, and Wnt/beta-catenin signaling pathways (12, 21, 30, 61, 62). Currently, there are limited studies regarding RGS proteins that were reported to regulate bone metabolism. Those RGS proteins are from subfamilies of R4, R12, RA, and GRK (**Table 1**) (30). The RGS proteins from other five RGS subfamilies have not been reported to regulate bone development and/or homeostasis. It is possible that those RGS proteins have no or lower expression in bone and/or no effect on bone. Additionally, the same RGS protein may contain various biological functions in different tissues and cells such as RGS12. Since the relationship between RGS proteins and bone is well-reviewed by Keinan et al. (30), this review mainly discussed the most recent findings on RGS proteins in bone.

Although extensive progress has been made in understanding how RGS proteins affect bone homeostasis, the following important questions remain to be determined: 1) subfamily members have similar protein domains and/or structure, so, what are the interactions of the same subfamily members? Are there mutually antagonistic or complementary functions between them? 2) What are the specific functions of RGS protein domains? Does the purified domain possess biological activity in bone? 3) What is the relationship between RGS proteins and osteoimmunology? Thus, the function of RGS proteins needs to be further studied using conditional or multiple RGS knockout animal models in specific bone cell lineages. Finally, due to the importance of RGS proteins in bone, development of agonists or antagonists of RGS proteins to specifically target particular bone cell lineages will provide us with new therapeutic candidates for bone-related diseases.

## Author Contributions

GY and SY conceived and wrote the manuscript. All authors contributed to the article and approved the submitted version.

## Funding

This work was supported by grants from the National Institute on Aging [NIA] (AG048388), the National Institute of Arthritis and Musculoskeletal and Skin Diseases [NIAMS] (AR066101), and Department of Defense office of the Congressionally Directed Medical Research Programs [CDMRP] (PR201467) to SY. This work was supported by grants from the Penn Center for Musculoskeletal Disorders [NIAMS] (P30-AR069619).

## Conflict of Interest

The authors declare that the research was conducted in the absence of any commercial or financial relationships that could be construed as a potential conflict of interest.

## Publisher’s Note

All claims expressed in this article are solely those of the authors and do not necessarily represent those of their affiliated organizations, or those of the publisher, the editors and the reviewers. Any product that may be evaluated in this article, or claim that may be made by its manufacturer, is not guaranteed or endorsed by the publisher.

## References

[1] Zaidi M. Skeletal Remodeling in Health and Disease. Nat Med (2007) 13:791–801. doi: 10.1038/nm1593 17618270

[2] Feng X McDonald JM. Disorders of Bone Remodeling. Annu Rev Pathol (2011) 6:121–45. doi: 10.1146/annurev-pathol-011110-130203 PMC3571087 20936937

[3] Manolagas SC. Birth and Death of Bone Cells: Basic Regulatory Mechanisms and Implications for the Pathogenesis and Treatment of Osteoporosis. Endocr Rev (2000) 21:115–37. doi: 10.1210/edrv.21.2.0395 10782361

[4] Dreier R. Hypertrophic Differentiation of Chondrocytes in Osteoarthritis: The Developmental Aspect of Degenerative Joint Disorders. Arthritis Res Ther (2010) 12:216. doi: 10.1186/ar3117 20959023 PMC2990991

[5] Rosenbaum DM Rasmussen SG Kobilka BK. The Structure and Function of G-Protein-Coupled Receptors. Nature (2009) 459:356–63. doi: 10.1038/nature08144 PMC3967846 19458711

[6] Tuteja N. Signaling Through G Protein Coupled Receptors. Plant Signal Behav (2009) 4:942–7. doi: 10.4161/psb.4.10.9530 PMC2801357 19826234

[7] Denis C Sauliere A Galandrin S Senard JM Gales C. Probing Heterotrimeric G Protein Activation: Applications to Biased Ligands. Curr Pharm Des (2012) 18:128–44. doi: 10.2174/138161212799040466 PMC3389521 22229559

[8] Xue C Hsueh YP Heitman J. Magnificent Seven: Roles of G Protein-Coupled Receptors in Extracellular Sensing in Fungi. FEMS Microbiol Rev (2008) 32:1010–32. doi: 10.1111/j.1574-6976.2008.00131.x PMC2998294 18811658

[9] Kimple AJ Bosch DE Giguere PM Siderovski DP. Regulators of G-Protein Signaling and Their Galpha Substrates: Promises and Challenges in Their Use as Drug Discovery Targets. Pharmacol Rev (2011) 63:728–49. doi: 10.1124/pr.110.003038 PMC3141876 21737532

[10] Jules J Yang S Chen W Li YP. Role of Regulators of G Protein Signaling Proteins in Bone Physiology and Pathophysiology. Prog Mol Biol Transl Sci (2015) 133:47–75. doi: 10.1016/bs.pmbts.2015.02.002 26123302 PMC4817727

[11] Masuho I Balaji S Muntean BS Skamangas NK Chavali S Tesmer JJG. A Global Map of G Protein Signaling Regulation by RGS Proteins. Cell (2020) 183:503–521.e519. doi: 10.1016/j.cell.2020.08.052 33007266 PMC7572916

[12] Sethakorn N Yau DM Dulin NO. Non-Canonical Functions of RGS Proteins. Cell Signal (2010) 22:1274–81. doi: 10.1016/j.cellsig.2010.03.016 PMC2893250 20363320

[13] Xie Z Chan EC Druey KM. R4 Regulator of G Protein Signaling (RGS) Proteins in Inflammation and Immunity. AAPS J (2016) 18:294–304. doi: 10.1208/s12248-015-9847-0 26597290 PMC4779105

[14] Senese NB Kandasamy R Kochan KE Traynor JR. Regulator of G-Protein Signaling (RGS) Protein Modulation of Opioid Receptor Signaling as a Potential Target for Pain Management. Front Mol Neurosci (2020) 13:5. doi: 10.3389/fnmol.2020.00005 32038168 PMC6992652

[15] Yuan G Yang S Gautam M Luo W Yang S. Macrophage Regulator of G-Protein Signaling 12 Contributes to Inflammatory Pain Hypersensitivity. Ann Transl Med (2021) 9:448. doi: 10.21037/atm-20-5729 33850845 PMC8039686

[16] Yuan G Yang S Yang S. Macrophage RGS12 Contributes to Osteoarthritis Pathogenesis Through Enhancing the Ubiquitination. Genes Dis (2021). doi: 10.1016/j.gendis.2021.08.005 PMC9293709 35873013

[17] Squires KE Montanez-Miranda C Pandya RR Torres MP Hepler JR. Genetic Analysis of Rare Human Variants of Regulators of G Protein Signaling Proteins and Their Role in Human Physiology and Disease. Pharmacol Rev (2018) 70:446–74. doi: 10.1124/pr.117.015354 PMC5989036 29871944

[18] O'Brien JB Wilkinson JC Roman DL. Regulator of G-Protein Signaling (RGS) Proteins as Drug Targets: Progress and Future Potentials. J Biol Chem (2019) 294:18571–85. doi: 10.1074/jbc.REV119.007060 PMC6901330 31636120

[19] Stewart A Fisher RA. Introduction: G Protein-Coupled Receptors and RGS Proteins. Prog Mol Biol Transl Sci (2015) 133:1–11. doi: 10.1016/bs.pmbts.2015.03.002 26123299

[20] Ren J Wei W Tan L Yang Q Lu Q Ding H. Inhibition of Regulator of G Protein Signaling 10, Aggravates Rheumatoid Arthritis Progression by Promoting NF-kappaB Signaling Pathway. Mol Immunol (2021) 134:236–46. doi: 10.1016/j.molimm.2021.03.024 33836352

[21] Yuan G Yang S Ng A Fu C Oursler MJ Xing L. RGS12 Is a Novel Critical NF-kappaB Activator in Inflammatory Arthritis. iScience (2020) 23:101172. doi: 10.1016/j.isci.2020.101172 32512384 PMC7281782

[22] Thirunavukkarasu K Halladay DL Miles RR Geringer CD Onyia JE. Analysis of Regulator of G-Protein Signaling-2 (RGS-2) Expression and Function in Osteoblastic Cells. J Cell Biochem (2002) 85:837–50. doi: 10.1002/jcb.10176 11968023

[23] Madrigal A Tan L Zhao Y. Expression Regulation and Functional Analysis of RGS2 and RGS4 in Adipogenic and Osteogenic Differentiation of Human Mesenchymal Stem Cells. Biol Res (2017) 50:43. doi: 10.1186/s40659-017-0148-1 29279050 PMC5742872

[24] Krieger NS Bushinsky DA. Metabolic Acidosis Regulates RGS16 and G Protein Signaling in Osteoblasts. Am J Physiol Renal Physiol (2021) 321:F424–30. doi: 10.1152/ajprenal.00166.2021 PMC8560403 34396788

[25] Shu B Zhao Y Zhao S Pan H Xie R Yi D. Inhibition of Axin1 in Osteoblast Precursor Cells Leads to Defects in Postnatal Bone Growth Through Suppressing Osteoclast Formation. Bone Res (2020) 8:31. doi: 10.1038/s41413-020-0104-5 32821442 PMC7424530

[26] Yan Y Tang D Chen M Huang J Xie R Jonason JH. Axin2 Controls Bone Remodeling Through the Beta-Catenin-BMP Signaling Pathway in Adult Mice. J Cell Sci (2009) 122:3566–78. doi: 10.1242/jcs.051904 PMC2746134 19737815

[27] Wang L Liu S Quarles LD Spurney RF. Targeted Overexpression of G Protein-Coupled Receptor Kinase-2 in Osteoblasts Promotes Bone Loss. Am J Physiol Endocrinol Metab (2005) 288:E826-34. doi: 10.1152/ajpendo.00422.2004 15585587

[28] Yang S Li YP. RGS10-Null Mutation Impairs Osteoclast Differentiation Resulting From the Loss of [Ca2+]i Oscillation Regulation. Genes Dev (2007) 21:1803–16. doi: 10.1101/gad.1544107 PMC1920174 17626792

[29] Iwai K Koike M Ohshima S Miyatake K Uchiyama Y Saeki Y. RGS18 Acts as a Negative Regulator of Osteoclastogenesis by Modulating the Acid-Sensing OGR1/NFAT Signaling Pathway. J Bone Miner Res (2007) 22:1612–20. doi: 10.1359/jbmr.070612 17576169

[30] Keinan D Yang S Cohen RE Yuan X Liu T Li YP. Role of Regulator of G Protein Signaling Proteins in Bone. Front Biosci (Landmark Ed) (2014) 19:634–48. doi: 10.2741/4232 PMC3966557 24389209

[31] Li L Zheng B Zhang F Luo X Li F Xu T. LINC00370 Modulates miR-222-3p-RGS4 Axis to Protect Against Osteoporosis Progression. Arch Gerontol Geriatr (2021) 97:104505. doi: 10.1016/j.archger.2021.104505 34450404

[32] Appleton CT James CG Beier F. Regulator of G-Protein Signaling (RGS) Proteins Differentially Control Chondrocyte Differentiation. J Cell Physiol (2006) 207:735–45. doi: 10.1002/jcp.20615 16489565

[33] Huang W Chung UI Kronenberg HM de Crombrugghe B. The Chondrogenic Transcription Factor Sox9 is a Target of Signaling by the Parathyroid Hormone-Related Peptide in the Growth Plate of Endochondral Bones. Proc Natl Acad Sci USA (2001) 98:160–5. doi: 10.1073/pnas.011393998 PMC14561 11120880

[34] Yang S Hao L McConnell M Zhou X Wang M Zhang Y. Inhibition of Rgs10 Expression Prevents Immune Cell Infiltration in Bacteria-Induced Inflammatory Lesions and Osteoclast-Mediated Bone Destruction. Bone Res (2013) 1:267–81. doi: 10.4248/BR201303005 PMC3994128 24761229

[35] Yang S Li YP. RGS12 is Essential for RANKL-Evoked Signaling for Terminal Differentiation of Osteoclasts *In Vitro* . J Bone Miner Res (2007) 22:45–54. doi: 10.1359/jbmr.061007 17042716 PMC3559086

[36] Ng AYH Li Z Jones MM Yang S Li C Fu C. Regulator of G Protein Signaling 12 Enhances Osteoclastogenesis by Suppressing Nrf2-Dependent Antioxidant Proteins to Promote the Generation of Reactive Oxygen Species. Elife (2019) 8:e42951. doi: 10.7554/eLife.42951 31490121 PMC6731062

[37] Li Z Liu T Gilmore A Gomez NM Fu C Lim J. Regulator of G Protein Signaling Protein 12 (Rgs12) Controls Mouse Osteoblast Differentiation *via* Calcium Channel/Oscillation and Galphai-ERK Signaling. J Bone Miner Res (2019) 34:752–64. doi: 10.1002/jbmr.3645 PMC7675783 30489658

[38] Yuan G Yang S Liu M Yang S. RGS12 is Required for the Maintenance of Mitochondrial Function During Skeletal Development. Cell Discov (2020) 6:59. doi: 10.1038/s41421-020-00190-w 32922858 PMC7459111

[39] Maruyama T Jiang M Abbott A Yu HI Huang Q Chrzanowska-Wodnicka M. Rap1b Is an Effector of Axin2 Regulating Crosstalk of Signaling Pathways During Skeletal Development. J Bone Miner Res (2017) 32:1816–28. doi: 10.1002/jbmr.3171 PMC5555789 28520221

[40] Wang K Xu C Xie X Jing Y Chen PJ Yadav S. Axin2+ PDL Cells Directly Contribute to New Alveolar Bone Formation in Response to Orthodontic Tension Force. J Dent Res (2022) 220345211062585. doi: 10.1177/00220345211062585 35001706 PMC9124907

[41] Yu HM Jerchow B Sheu TJ Liu B Costantini F Puzas JE. The Role of Axin2 in Calvarial Morphogenesis and Craniosynostosis. Development (2005) 132:1995–2005. doi: 10.1242/dev.01786 15790973 PMC1828115

[42] Spurney RF Flannery PJ Garner SC Athirakul K Liu S Guilak F. Anabolic Effects of a G Protein-Coupled Receptor Kinase Inhibitor Expressed in Osteoblasts. J Clin Invest (2002) 109:1361–71. doi: 10.1172/JCI14663 PMC150976 12021252

[43] Hinton RJ Jing Y Jing J Feng JQ. Roles of Chondrocytes in Endochondral Bone Formation and Fracture Repair. J Dent Res (2017) 96:23–30. doi: 10.1177/0022034516668321 27664203 PMC5347428

[44] Lefebvre V Dvir-Ginzberg M. SOX9 and the Many Facets of its Regulation in the Chondrocyte Lineage. Connect Tissue Res (2017) 58:2–14. doi: 10.1080/03008207.2016.1183667 27128146 PMC5287363

[45] Luo J Yang Z Ma Y Yue Z Lin H Qu G. LGR4 is a Receptor for RANKL and Negatively Regulates Osteoclast Differentiation and Bone Resorption. Nat Med (2016) 22:539–46. doi: 10.1038/nm.4076 27064449

[46] Komarova SV Pereverzev A Shum JW Sims SM Dixon SJ. Convergent Signaling by Acidosis and Receptor Activator of NF-kappaB Ligand (RANKL) on the Calcium/Calcineurin/NFAT Pathway in Osteoclasts. Proc Natl Acad Sci USA (2005) 102:2643–8. doi: 10.1073/pnas.0406874102 PMC548977 15695591

[47] Masi L Brandi ML. Calcitonin and Calcitonin Receptors. Clin Cases Miner Bone Metab (2007) 4:117–22. PMC2781237 22461211

[48] Yuan G Fu C Yang ST Yuh DY Hajishengallis G Yang S. RGS12 Drives Macrophage Activation and Osteoclastogenesis in Periodontitis. J Dent Res (2021) 101(4):448–57. doi: 10.1177/00220345211045303 PMC8935576 34796776

[49] Larminie C Murdock P Walhin JP Duckworth M Blumer KJ Scheideler MA. Selective Expression of Regulators of G-Protein Signaling (RGS) in the Human Central Nervous System. Brain Res Mol Brain Res (2004) 122:24–34. doi: 10.1016/j.molbrainres.2003.11.014 14992813

[50] Koh J Dar M Untch BR Dixit D Shi Y Yang Z. Regulator of G Protein Signaling 5 is Highly Expressed in Parathyroid Tumors and Inhibits Signaling by the Calcium-Sensing Receptor. Mol Endocrinol (2011) 25:867–76. doi: 10.1210/me.2010-0277 PMC3082322 21393447

[51] Balenga N Koh J Azimzadeh P Hogue J Gabr M Stains JP. Parathyroid-Targeted Overexpression of Regulator of G-Protein Signaling 5 (RGS5) Causes Hyperparathyroidism in Transgenic Mice. J Bone Miner Res (2019) 34:955–63. doi: 10.1002/jbmr.3674 PMC8210536 30690792

[52] Hay E Laplantine E Geoffroy V Frain M Kohler T Muller R. N-Cadherin Interacts With Axin and LRP5 to Negatively Regulate Wnt/beta-Catenin Signaling, Osteoblast Function, and Bone Formation. Mol Cell Biol (2009) 29:953–64. doi: 10.1128/MCB.00349-08 PMC2643807 19075000

[53] Ransom RC Hunter DJ Hyman S Singh G Ransom SC Shen EZ. Axin2-Expressing Cells Execute Regeneration After Skeletal Injury. Sci Rep (2016) 6:36524. doi: 10.1038/srep36524 27853243 PMC5113299

[54] Yang S Li YP Liu T He X Yuan X Li C. Mx1-Cre Mediated Rgs12 Conditional Knockout Mice Exhibit Increased Bone Mass Phenotype. Genesis (2013) 51:201–9. doi: 10.1002/dvg.22373 PMC3908791 23349096

[55] Yuan X Cao J Liu T Li YP Scannapieco F He X. Regulators of G Protein Signaling 12 Promotes Osteoclastogenesis in Bone Remodeling and Pathological Bone Loss. Cell Death Differ (2015) 22:2046–57. doi: 10.1038/cdd.2015.45 PMC4816106 25909889

[56] Cekici A Kantarci A Hasturk H Van Dyke TE. Inflammatory and Immune Pathways in the Pathogenesis of Periodontal Disease. Periodontol 2000 (2014) 64:57–80. doi: 10.1111/prd.12002 24320956 PMC4500791

[57] Mbalaviele G Novack DV Schett G Teitelbaum SL. Inflammatory Osteolysis: A Conspiracy Against Bone. J Clin Invest (2017) 127:2030–9. doi: 10.1172/JCI93356 PMC5451216 28569732

[58] Zhang K He S Dai Z Cao L Yue S Bai Y. Axin 1 Knockdown Inhibits Osteoblastic Apoptosis Induced by Porphyromonas Gingivalis Lipopolysaccharide. Arch Oral Biol (2020) 112:104667. doi: 10.1016/j.archoralbio.2020.104667 32092441

[59] Hu X Tang J Zeng G Hu X Bao P Wu J. RGS1 Silencing Inhibits the Inflammatory Response and Angiogenesis in Rheumatoid Arthritis Rats Through the Inactivation of Toll-Like Receptor Signaling Pathway. J Cell Physiol (2019) 234:20432–42. doi: 10.1002/jcp.28645 31012109

[60] Schiff ML Siderovski DP Jordan JD Brothers G Snow B De Vries L. Tyrosine-Kinase-Dependent Recruitment of RGS12 to the N-Type Calcium Channel. Nature (2000) 408:723–7. doi: 10.1038/35047093 11130074

[61] Lee JK Chung J McAlpine FE Tansey MG. Regulator of G-Protein Signaling-10 Negatively Regulates NF-kappaB in Microglia and Neuroprotects Dopaminergic Neurons in Hemiparkinsonian Rats. J Neurosci (2011) 31:11879–88. doi: 10.1523/JNEUROSCI.1002-11.2011 PMC3326398 21849548

[62] Willard MD Willard FS Li X Cappell SD Snider WD Siderovski DP. Selective Role for RGS12 as a Ras/Raf/MEK Scaffold in Nerve Growth Factor-Mediated Differentiation. EMBO J (2007) 26:2029–40. doi: 10.1038/sj.emboj.7601659 PMC1852785 17380122

